# The impact of TP53 activation and apoptosis in primary hereditary microcephaly

**DOI:** 10.3389/fnins.2023.1220010

**Published:** 2023-06-28

**Authors:** Giorgia Iegiani, Alessia Ferraro, Gianmarco Pallavicini, Ferdinando Di Cunto

**Affiliations:** ^1^Department of Neuroscience ‘Rita Levi Montalcini’, University of Turin, Turin, Italy; ^2^Neuroscience Institute Cavalieri Ottolenghi, Turin, Italy

**Keywords:** neurodevelopment, microcephaly, DNA damage, TP53, cell death, asymmetric division, mitosis, neural precursor

## Abstract

Autosomal recessive primary microcephaly (MCPH) is a constellation of disorders that share significant brain size reduction and mild to moderate intellectual disability, which may be accompanied by a large variety of more invalidating clinical signs. Extensive neural progenitor cells (NPC) proliferation and differentiation are essential to determine brain final size. Accordingly, the 30 MCPH loci mapped so far (MCPH1-MCPH30) encode for proteins involved in microtubule and spindle organization, centriole biogenesis, nuclear envelope, DNA replication and repair, underscoring that a wide variety of cellular processes is required for sustaining NPC expansion during development. Current models propose that altered balance between symmetric and asymmetric division, as well as premature differentiation, are the main mechanisms leading to MCPH. Although studies of cellular alterations in microcephaly models have constantly shown the co-existence of high DNA damage and apoptosis levels, these mechanisms are less considered as primary factors. In this review we highlight how the molecular and cellular events produced by mutation of the majority of MCPH genes may converge on apoptotic death of NPCs and neurons, via TP53 activation. We propose that these mechanisms should be more carefully considered in the alterations of the sophisticated equilibrium between proliferation, differentiation and death produced by MCPH gene mutations. In consideration of the potential druggability of cell apoptotic pathways, a better understanding of their role in MCPH may significantly facilitate the development of translational approaches.

## 1. Introduction

Microcephaly is a rare condition in which an individual’s occipital-frontal head circumference (OFC) is reduced more than two (in microcephaly) or three (in severe microcephaly) standard deviations below the mean for a given sex, age, and ethnicity (Von der Hagen et al., 2014). Microcephaly occurs in 1.5 to 8.7 out of every 10,000 births in Europe and the United States, respectively (Cragan et al., 2016; Morris et al., 2016). However, it’s worth noting that 15 to 20% of children who experience developmental delay also have microcephaly (Watemberg et al., 2002; Aggarwal et al., 2013).

The effects of microcephaly can vary widely, ranging from mild to severe. The most frequent symptoms are intellectual disability, developmental delay, and neurological problems but children with microcephaly may also experience seizures, difficulties with balance and coordination, and impaired vision or hearing (Woods and Parker, 2013; Devakumar et al., 2018).

Microcephaly can be classified as primary or secondary based on the timing of its onset (Zaqout et al., 2017; Zaqout and Kaindl, 2021). Primary microcephaly is present at birth and is characterized by a decrease of neurons’ number. Conversely, secondary microcephaly arises after birth and impacts more on dendritic complexity, formation of synaptic contacts and myelination (Woods, 2004; Dupuis et al., 2015; Zaqout and Kaindl, 2021). The causes of microcephaly can be categorized into two main groups: genetic and environmental. Environmental factors leading to microcephaly include congenital infections affecting the brain, exposure to radiation, toxins or teratogenic agents during pregnancy (e.g., fetal alcohol syndrome), and hypoxic–ischemic injury that occurs either before or during birth (Mochida and Walsh, 2001). Many viral infections such as Cytomegalovirus, Influenza, Herpes Simplex and Zika, as well as parasitic infections like Toxoplasma gondii have been linked to primary microcephaly (Devakumar et al., 2018). To differentiate between primary microcephaly caused by genetic defects and congenital microcephaly induced by environmental factors, primary hereditary microcephaly (MCPH) subclass was defined (Woods et al., 2005).

**Figure 1:**
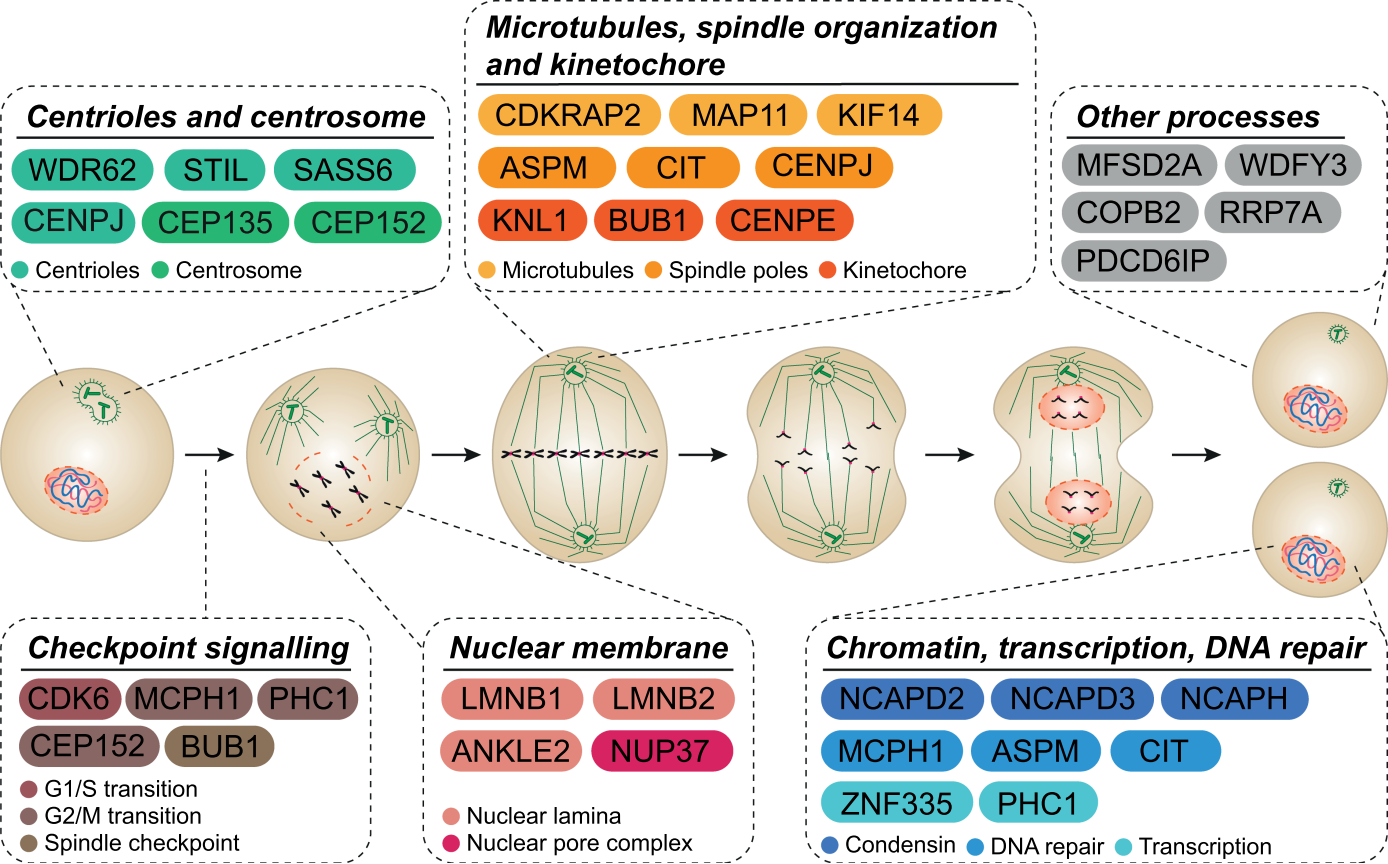
MCPH cellular functions. MCPH-associated proteins are involved in many cellular functions throughout the cell cycle. Several proteins act across more than one functional pathway (i.e., mitotic spindle orientation and DNA repair).

MCPH is a syndrome that occurs when single locus mutations lead to reduced brain size. To date, 30 different genes have been identified as causes of MCPH (Figure 1; Faheem et al., 2015; Zaqout et al., 2017). MCPH genes are expressed in all proliferating cell types and, during cortical neurogenesis, are expressed at high levels in the ventricular zone (VZ), the primary germinal zone of the cerebral cortex, which is the most affected structure in microcephaly patients (Bond and Woods, 2006; Manzini and Walsh, 2011).

## 2. Normal corticogenesis

During the early stages of neurogenesis, neuroepithelial cells rapidly divide to increase the stem cell pool and densify the wall of the neural tube (Rakic, 1995). By the midstage of neurogenesis, NPCs can be divided into two types: apical and basal progenitors (Florio and Huttner, 2014). Apical progenitor cells lie in the VZ while basal progenitor cells are found in the subventricular zone (SVZ), which is adjacent to the VZ. The VZ contains several types of progenitors, including apical radial glial cells (aRGCs), apical intermediate progenitors, and subapical progenitors (Schultze and Korr, 1981; Malatesta et al., 2003; Götz and Huttner, 2005; Pilz et al., 2013; Tyler and Haydar, 2013). aRGCs are the predominant class of progenitors in the VZ (Noctor et al., 2002) and, at the beginning, undergo several rounds of symmetric or proliferative division to generate two identical daughter cells, thus expanding the number of progenitors. Subsequently aRGCs undergo asymmetric division, producing one stem cell and one fate-restricted daughter cell, i.e., basal progenitor or a postmitotic neuron (Götz and Huttner, 2005; Taverna et al., 2014).

In primates, basal progenitors include intermediate progenitors (IPs) and a second cell type called basal radial glial cells (bRGCs) (Fietz et al., 2010; Hansen et al., 2010). IPs can undergo one to two additional rounds of division to generate more IPs or commit to terminal division, generating two daughter neurons (Haubensak et al., 2004; Wu et al., 2005). bRGCs are highly neurogenic, arise from the division of aRGCs and are involved in development of cortical folds or gyri (Penisson et al., 2019). Lastly, toward the end of neurogenesis, RGCs participate in generation of glial cells, which are essential for proper neuronal function (Qian et al., 2000; Jäkel and Dimou, 2017). After neurogenic divisions, post-mitotic neurons migrate radially and progressively settle in the more superficial cortical layers (inside-out migration; Rakic, 1971; McConnell and Kaznowski, 1991). By the time of birth, all the neurons that contribute to cortical architecture and function have been produced and most NPCs have been depleted. Decreased proliferation, premature commitment to neurogenic division or the death of progenitors and/or their progeny may lead to a significantly depleted NPC pool, resulting in fewer neocortical cells (Phan and Holland, 2021).

## 3. Microcephaly genes are involved in cell cycle or cell division regulation

MCPH genes are expressed in all proliferating cell types but are selectively required in NPC. The biological basis of this functional specificity is only partially understood (Faheem et al., 2015; Zhou et al., 2020). Due to their strong expression in proliferating cells, investigation of MCPH genes’ biological role first focused on the analysis of cell cycle or mitosis.

A set of MCPH genes (MCPH1, CEP152, PHC1) participate in control of the G2-M checkpoint (Morris-Rosendahl and Kaindl, 2015), that prevents cells from entering mitosis in case of damaged or incomplete DNA replication. MCPH1 deficiency prevents the recruitment of checkpoint kinase 1 (Chk1) to centrosomes, leading to premature cyclin activation and early mitotic entry, uncoupled from centrosome cycle (Gruber et al., 2011). Cell cycle analysis in CEP152 knockdown cells suggested that CEP152 deficiency delays S-phase entry. Furthermore, fewer cells progress to the G2/M phase and an increased proportion of cells stayed in G0/G1 (Kalay et al., 2011). PHC1 regulates cell cycle by interacting with geminin, which has an established role in cell cycle control (Luo and Kessel, 2004).

Another set of MCPH genes (ASPM, KIF14, MAP11, CENPE, CENPJ, KNL1, CDK5RAP2, CIT and BUB1) is involved in mitotic spindle organization and microtubule dynamics, important for proper segregation of chromosomes in daughter cells (Morris-Rosendahl and Kaindl, 2015; Pallavicini et al., 2019; Phan and Holland, 2021; Iegiani et al., 2021a,b). Mutations in these genes increase the frequency of cytokinesis failure and the generation of polyploid progeny (Moawia et al., 2017; Bianchi et al., 2017b).

In addition, some microcephaly genes are required for centriole duplication (CENPJ, STILL, WDR62) and centriole assembly (CEP152, CEP135, STIL, SASS6) (Zaqout and Kaindl, 2021). Production of the correct number of centrioles and their correct assembly is fundamental for proper distribution of chromosomes to daughter cells, avoiding spindle instability and mitotic delay or arrest at metaphase checkpoint (Lizarraga et al., 2010; Vitale et al., 2011; Novorol et al., 2013; Chen et al., 2014). Mutations in genes encoding MCPH centrosome proteins alter the maturation and the number of centrosomes (Rodrigues-Martins et al., 2007; Yabe et al., 2007; Megraw et al., 2011; Hussain et al., 2013), which may increase the ratio of asymmetric divisions (Pfaff et al., 2007; Vitale et al., 2011; McIntyre et al., 2012; Novorol et al., 2013). Enhanced NPC asymmetric division contributes to the microcephalic phenotype by depleting the neural stem cell pool (Pfaff et al., 2007; Gruber et al., 2011; Ding et al., 2019; Zhang et al., 2019).

Although dysregulation of the balance between symmetric and asymmetric division is commonly considered crucial for MCPH, it has been reported that cell fate alterations correlated with asymmetric divisions could be more dependent on cell cycle dysregulation than on morphological asymmetry (Taverna et al., 2014; Capecchi and Pozner, 2015). Timing of the cell cycle is fundamental for proper division (Calegari and Huttner, 2003; Calegari et al., 2005). G1-phase length defines if cells progress to S-phase, enter quiescence or differentiate (Capecchi and Pozner, 2015). Forced shortening of the NPC cell cycle promotes expansion of neural progenitors through symmetric proliferative cell divisions (Lange et al., 2009; Pilaz et al., 2009; Artegiani et al., 2011; Nonaka-Kinoshita et al., 2013). Conversely, artificial lengthening of mitosis leads to increased frequency of asymmetric divisions and neural commitment (Pilaz et al., 2016).

Reduced proliferation, symmetric and asymmetric division dysregulation after MCPH genes loss may all decrease expansion rates in brain development but may only provide a partial explanation for the selective consequences of MCPH gene mutations and for the severity of the corresponding phenotypes.

## 4. Loss of MCPH genes leads to increased cell death in the developing CNS

**Figure 2:**
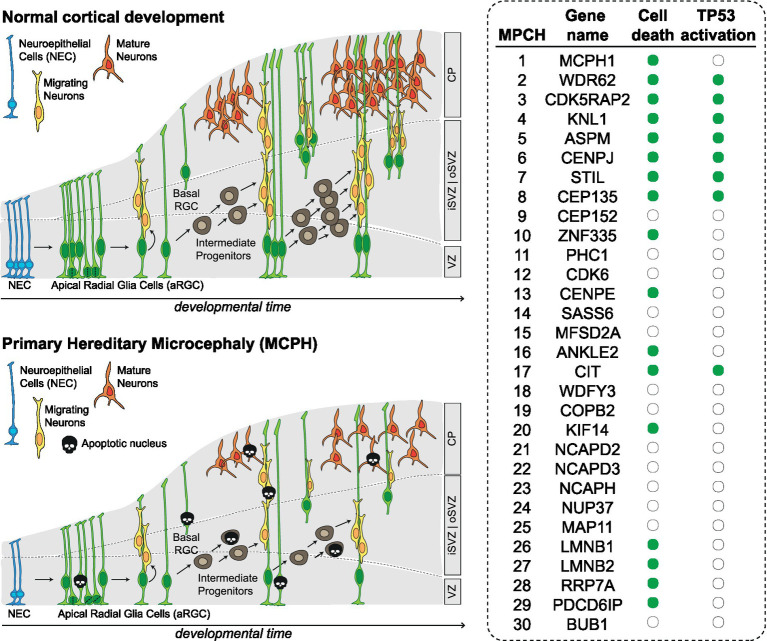
Altered neurodevelopment in MCPH. Scheme depicting the main types of progenitor cells and their lineage relationships during normal development of cerebral cortex and in MCPH. The table on the right shows for which MCPH genes there is evidence of apoptosis and TP53 activation (green dot). CP, cortical plate; iSVZ, inner subventricular zone; oSVZ, outer subventricular zone VZ, ventricular zone; NEC, neuroepithelial cells; aRGC, apical radial glia cells; bRGC, basal radial glia cells.

Programmed cell death contributes to CNS development by regulating cell number, eliminating signaling centers, allowing proper spacing, positioning and avoiding mis-specification (Yamaguchi and Miura, 2015). However, most of the analyzed MCPH models are characterized by increased cell death, far exceeding physiological levels (Figure 2). For example, MCPH1-deficient mice show increased percentage of apoptotic cells, both under basal conditions and after ionizing radiation (Zhou et al., 2013). Similarly, ASPM deficiency increases apoptosis during development both in mouse and zebrafish models (Novorol et al., 2013; Williams et al., 2015). CITK loss leads to massive apoptosis in rodents (Di Cunto et al., 2000; Sarkisian et al., 2002; Sgrò et al., 2016; Bianchi et al., 2017b). Apoptosis is observed after KNL1 loss (Shi et al., 2019) and after CENPJ loss both in mice and human cerebral organoids (Lin et al., 2020; An et al., 2022). Mutations in LMNB1 and LMNB2 lead to increased apoptosis in the developing neocortex and neurons (Coffinier et al., 2011; Chen et al., 2019). Mutation of WDR62, whose product is functionally required for spindle pole organization, leads to apoptosis in the developing mouse neocortex and in human cerebral organoids (Chen et al., 2014; Zhang et al., 2019). Similarly, mutations in the CDK5RAP2, also involved in mitotic spindle orientation, lead to apoptosis in the developing neocortex (González-Martínez et al., 2021). Loss of centriolar proteins like STIL and CEP135 also leads to apoptosis in neural progenitors (Novorol et al., 2013; González-Martínez et al., 2021); CENPE loss lead to apoptosis in cell lines related to neural progenitors (Iegiani et al., 2021a,b). Mutations in KIF14, encoding a microtubule motor protein, lead to apoptosis in mice (Fujikura et al., 2013) as well as in patient derived cells (Moawia et al., 2017). Inactivation of transcription factor ZNF335 induces cell death (Yang et al., 2012). In Drosophila models, loss of the orthologues of mitotic nuclear envelope reassembly regulator ANKLE2 (Yamamoto et al., 2014), as well as of the rRNA processing and ribosomal small subunit assembly protein RRP7A, have been associated to increased apoptosis (Farooq et al., 2020). Finally, loss of PDCD6IP, a multifunctional protein involved in endosomal trafficking, cytokinesis and maintenance of tight junction integrity, also leads to massive apoptosis in developing cortex (Trioulier et al., 2004; Laporte et al., 2017).

All together, these data show that inactivation of MCPH genes is associated with sensible increase of NPC cell death regardless of molecular functions of the involved genes. Apoptosis has not been assessed in the developing CNS for MCPH genes CEP152, PHC1, CDK6, SASS6, MFSD2A, WDFY3, COPB2, NCAPD2, NCAPD3, NCAPH, NUP37, MAP11, BUB1, which are involved in centriole biogenesis, chromatin and transcriptional regulation, cell cycle progression and kinetochore assembly. Since apoptosis can result from defects in the cellular mechanisms in which these proteins are involved, it seems reasonable to predict that also these mutations can lead to increased cell death in the developing nervous system.

## 5. Cell death generated by loss of MCPH genes is mostly TP53-dependent

The nuclear transcription factor TP53 regulates several major cellular functions including gene transcription, DNA synthesis, DNA repair, cell cycle regulation, senescence, and cell death (Hafner et al., 2019). Several genes associated with MCPH have been shown to induce activation of TP53 (Figure 2). For example, CDK5RAP2 deficiency induces TP53 expression in mice neural progenitors (González-Martínez et al., 2021) and in patient derived cells (Wang et al., 2021). Mutations in KNL1 lead to activation of TP53 and TP53 target genes in KNL1 conditional knock-out brain (Shi et al., 2019). Mutations in CENPJ lead to TP53 activation both in mice and human cerebral organoids (Lin et al., 2020; An et al., 2022). Similarly, ASPM, STIL, WDR62 and CITK mutations have been shown to activate the TP53 pathway (Novorol et al., 2013; Williams et al., 2015; Bianchi et al., 2017b; Pallavicini et al., 2018). In addition, mutations in CENPJ and CDK5RAP2 can lead to centrosome abnormalities, which can in turn activate the TP53 pathway and contribute to the development of microcephaly (Phan et al., 2021).

TP53 is a major regulator of apoptosis (Aubrey et al., 2018) and is probably the main driver of this process in MCPH (Figure 2). This is supported by the fact that TP53 co-deletion largely rescues cell loss phenotypes, in mice and other pre-clinical models generated by MCPH genes loss. KNL1/TP53 conditional double mutant mice cortical size is partially restored and apoptosis occurred at significantly lower levels (Shi et al., 2019), when compare with isolated KNL1 knockout. Blocking TP53 activity in STIL, WDR62 or ASPM mutant zebrafish also led to reduced apoptosis (Novorol et al., 2013). Co-deletion of TP53 with ASPM in mice restored cerebellar growth and reduced apoptosis (Williams et al., 2015). Similarly, co-deletion of TP53 rescues radial glia progenitors from apoptosis induced by CENPJ or CEP135 loss (Lin et al., 2020; González-Martínez et al., 2021). Finally, in CITK/TP53 mutant mice, neural progenitors’ and neurons’ cell death is dramatically reduced; notably, the perinatal lethality that characterize this model was totally rescued, while clinical and neuroanatomical phenotypes were significantly improved, despite the persistence of a huge proportion of binucleated or polyploid cells (Bianchi et al., 2017b). These data highlight the crucial relevance of TP53 in contributing to neural cells’ loss that characterizes MCPH. The pathogenic effect of its activation in MCPH can be further potentiated by cell cycle arrest, the other prominent outcome of TP53 activation (Chen, 2016).

## 6. Mechanisms of TP53 activation in MCPH: DNA damage and beyond

Tight maintenance of genomic integrity appears to be an essential prerequisite for the development and function of the central nervous system (McKinnon, 2013). During the expansion of neural progenitor cells, a large number of DNA breaks is produced during DNA synthesis and mitosis, probably as a result of DNA replication stress induced by the high proliferative activity (McKinnon, 2013). Proper repair of these lesions is fundamental (Hakem, 2008). A large body of evidence has highlighted TP53 as the toughest ‘guardian of the genome’, capable to prevent genome instability by halting proliferation, promoting DNA repair or inducing cell death in many situations of genome imbalance and/or DNA damage (Hernández Borrero and El-Deiry, 2021). Loss of proteins strongly involved in the DNA damage response leads to TP53-dependent apoptosis. For example, conditional INO80 deletion from cortical NPCs impairs DNA double-strand break repair, triggering TP53-dependent apoptosis and microcephaly. INO80 is involved in nucleosome remodeling and histone variant exchange, and TP53 co-deletion extensively rescues INO80 conditional knockout phenotypes (Keil et al., 2020). Another example is BRCA2, a protein necessary for homologous recombination-mediated DNA repair. Conditional BRCA2 knockout affects neurogenesis, particularly during embryonic and postnatal neural development, while TP53 co-deletion largely restores brain histology (Frappart et al., 2007). One last case is represented by LIG4, that mediates DNA damage repair via non-homologous end joining. LIG4 knockout leads to embryonic lethality and massive neuronal apoptosis in mice, which can be recovered by TP53 co-deletion (Frank et al., 2000).

On this basis, it is not surprising that accumulation of DNA damage has been documented in many MCPH models and that the function of several MCPH genes is directly linked to DNA repair and genomic stability (Bianchi et al., 2018). The best example in this sense is probably MCPH1, encoding a centrosomal protein with three BRCA1 C-Terminus (BRCT) domains, involved in DNA repair, genomic stability, and chromatin remodeling (Liu et al., 2016). MCPH1 is a mediator of ATM and ATR pathways in response to DNA damage and co-localizes with numerous proteins involved in the DNA damage response (DDR) such as γH2AX, MDC1, 53BP1, RAD17, and RPA34 upon ionizing radiation or UV treatment (Wood et al., 2007; Liang et al., 2010). Moreover, The N-terminal BRCT domain interacts with the chromatin remodeling complex SWI/SNF in DNA repair (Peng et al., 2009). Consistent with its role in the DNA damage response, MCPH1-deficient neuronal progenitors are hypersensitive to ionizing radiation during neurogenesis. Moreover, deletion of MCPH1 compromises homologous recombination repair and induces genomic instability (Zhou et al., 2013). Some MCPH genes originally associated with other cellular functions have lately been involved in DNA repair processes. ASPM, the most frequently mutated among MPCH genes, is best known for its capability to focus microtubules minus ends at spindle pole bodies (Tungadi et al., 2017). However, ASPM levels are influenced by irradiation and, more importantly, ASPM knockdown impairs DNA double-strand break repair (Kato et al., 2011). Specifically, it has recently been found that ASPM can be recruited to DNA damage sites and is required for efficient homologous recombination repair (Xu et al., 2021). Moreover, ASPM is involved in stabilization of stalled fork in response to replication stress (Wu et al., 2022). Accordingly, ASPM disruption leads to increased DNA damage in cerebellar progenitor cells (Williams et al., 2015). Another gene that has been linked in recent years to DNA repair is CITK, best known for its involvement in cytokinesis (Madaule et al., 2000; D’Avino, 2017; Bianchi et al., 2017a). CITK loss induces DNA damage accumulation and chromosomal instability in both mammals and Drosophila. CITK-deficient cells display increased sensitivity to ionizing radiation, and defective recovery from radiation-induced DNA lesions (Bianchi et al., 2017b; Pallavicini et al., 2020; Boda et al., 2022). In particular, CITK binds RAD51 and is involved in its recruitment to DNA double-strand breaks (Bianchi et al., 2017a,b; Pallavicini et al., 2018), as well as in homologous recombination-dependent DNA repair (Pallavicini et al., 2020).

Inactivation of many other MPCH genes results in DNA damage accumulation even though they have not been implicated directly in DNA repair. KNL1, part of the KNL-1/Mis12/Ndc80 complex (KMN), is needed for proper kinetochore assembly, checkpoint functioning and spindle assembly checkpoint signaling (Caldas and DeLuca, 2014). Loss of KNL1 leads to DNA damage accumulation in NPC located in VZ and SVZ (Shi et al., 2019). Disruption of CENPJ, a regulator of centriole biogenesis, leads to genomic instability without altering ATR and ATM-dependent DNA damage signaling (McIntyre et al., 2012). Similarly, CEP152, a regulator of centriole duplication, has been described as regulator of genomic integrity and cellular response to DNA damage (Kalay et al., 2011). Impaired CEP152 function leads to increased H2AX phosphorylation and genomic instability (Kalay et al., 2011). The kinetochore motor protein CENPE, important in chromosome congression, spindle microtubule capture at kinetochores and spindle assembly checkpoint, also leads to DNA damage accumulation in in cell lines related to neural progenitors (Iegiani et al., 2021a,b). Mutations in LMNB1 and LMNB2, components of the nuclear lamina, cause DNA damage in neurons (Chen et al., 2019). The high prevalence of DNA damage accumulation after MCPH genes’ mutation suggest that many of them could play a direct role in avoiding chromosomal breaks or facilitating DNA repair. This could be reasonably predicted for PHC1, a gene involved in transcriptional regulation, whose loss leads not only to DNA damage accumulation, but also to decreased DNA repair after irradiation (Awad et al., 2013).

Besides DNA damage accumulation, other mechanisms may be responsible for or may contribute to the activation of TP53 in MCPH. Cytokinesis failure, characteristic of some microcephaly models (Di Cunto et al., 2000; Higgins et al., 2010; Harding et al., 2016; Perez et al., 2019; Reilly et al., 2019; Tedeschi et al., 2020; Little et al., 2021) has been reported to stabilize TP53 by engaging the Hippo pathway (Ganem et al., 2014). The unfolded protein response (UPR), which has been documented in specific genetic microcephaly syndromes (Laguesse et al., 2015; Passemard et al., 2019; Terabayashi and Hashimoto, 2021; Liu et al., 2023), and could be generally engaged by aneuploidy (Zanetti et al., 2016), may activate TP53 through NFkB engagement (Lin et al., 2012). Finally, disruption of centrosomal function, which may be produced by many MCPH mutations (Robinson et al., 2020), may stimulate P53 activity through the 53BP1-USP28 axis (Cuella-Martin et al., 2016; Meitinger et al., 2016).

## 7. TP53-independent determinants of MCPH revealed by co-deletion experiments

The mentioned studies highlight TP53 as a crucial crossroad of the events activated by mutation of MCPH genes, raising the possibility that TP53 activation may be a necessary event in most cases. On the other hand, co-deletion experiments also show that TP53 engagement may not be sufficient to produce the MCPH full phenotypic spectrum.

Although co-deletion of TP53 rescued perinatal lethality the massive apoptosis in CITK null mice, brain size and architecture were only partially restored (Bianchi et al., 2017a,b). Moreover, high throughput analysis of gene expression revealed a TP53-independent engagement of proliferation-suppressing pathways in the developing cerebellum of CITK/TP53 double knockouts (Bianchi et al., 2017a,b). Loss of centrosomal protein CEP135, responsible for MCPH8, results in centriole duplication defects, TP53 activation, and NPCs cell death (González-Martínez et al., 2021). TP53 ablation in a CEP135-deficient background prevents cell death but not MCPH, leading to subcortical heterotopias similar to those seen in MCPH8 patients (González-Martínez et al., 2021). Homozygous CENPJ deletion in the central nervous system causes dramatic apoptosis that severely disrupts embryonic brains (Lin et al., 2020). Microcephalic brains with reduced apoptosis are detected in conditional CENPJ knockout mice that lose only one allele of TP53, while simultaneous removal of TP53 and CENPJ fully rescues RGP death. Nevertheless, TP53 deletion has no effects on the other phenotypes that characterize this model, including cilia loss, RGP mislocalization, junctional integrity disruption, massive heterotopia and severe cerebellar hypoplasia that (Lin et al., 2020). Intriguingly, despite apoptosis elimination, conditional CENPJ/TP53 double knock-out animals have smaller brain than conditional CENPJ knock-out mice. These results suggest that lack of TP53-dependent adaptation to centriole defects in NPCs may lead to architectural defects if chromosomally unstable cells are not eliminated during brain development. Systematic analysis of STIL, ASPM and WDR62 in zebrafish through morpholino-induced ablation result in a marked reduction in head and eye size (Novorol et al., 2013). Live imaging studies made possible by this model revealed a dramatic rise in the fraction of proliferating cells, caused by failure of progression through prometaphase, accompanied by strongly increased levels of apoptosis. Blocking TP53 in this context led to a partial rescue of apoptosis and cell number, but had no effect on the mitotic phenotype (Novorol et al., 2013). This implies that, especially in the retina, changes in the ratio between asymmetric and symmetric divisions may impact on eye size independently of apoptosis and cell cycle block derived from TP53 activation (Malicki, 2004; Zigman et al., 2005). This is supported by the fact that several pathways are associated to the control of the mitotic spindle orientation and subsequent division angle in the retina, such as Notch signaling (Balenci and van der Kooy, 2014) and Laminin β2 Chain (Serjanov et al., 2018). Changes in mitotic spindle orientation in retinal progenitors leads to defects in cell fate specification and proliferation (Zigman et al., 2005). All together, these findings and data from retina studies strongly suggest that other pathways activated in parallel with TP53 activation may contribute in decreasing neural cell generation and increase neural cell death in MCPH syndromes.

## 8. Conclusion

After identification and functional characterization of the first causal genes, MCPH has long been considered a disorder primarily caused by specific alterations of the delicate balance between symmetric and asymmetric divisions that characterize cell fate determination in normal brain development. The coexistence of DNA damage and apoptosis, revealed by more in depth analysis, has progressively underscored the crucial role of TP53. Not surprisingly, the many mechanisms that may trigger the activation of this fundamental protein, especially DNA damage accumulation, have turned out to be highly relevant in the context of genetic microcephaly. On this basis, it can be tempting to speculate that explaining the link between a particular MCPH gene loss and TP53 engagement may be one of the most critical aspects for understanding the pathogenesis of the different clinical syndromes. Understanding the subtleties of TP53 regulation and action in NPCs and neurons is also relevant to explain the strong tissue-specificity of MCPH genes’ loss.

Although double knockout studies clearly reveal that other pathways may cooperate with TP53 in the production of full-blown phenotypes, the constant involvement of this cellular hub highlights at least one common druggable player (Pani et al., 2002; Strom et al., 2006). Several therapeutic strategies to inhibit TP53 have been described, such as Pifithrin-α (Sohn et al., 2009) and trifluoperazine (Taylor et al., 2020) that suppress TP53 mRNA transactivation and translation, respectively. Another option is represented by the use of short interfering RNA technology to silence TP53 (Ubby et al., 2019). Finally, besides direct TP53 inhibition, other strategies could aim at targeting cellular process that are altered when TP53 is hyperactivated such as inhibition of TGF-β signaling or upregulation of mTOR signaling (Tsai et al., 2021). The potential risks of developing translational strategies based on T53 inactivation underscore the relevance of better understanding the specificities of TP53 downstream pathways’ engagement in MCPH.

## Author contributions

GI, GP, and FDC contributed to conception and design of the study. GI, AF, and GP wrote the first draft of the manuscript. GI made figures. All authors contributed to manuscript revision, read, and approved the submitted version.

## Funding

This work was mainly supported by Associazione Italiana per la Ricerca sul Cancro (AIRC) with grant IG23341 to FDC. GP was supported by a fellowship from AIRC. The contribution of the University of Torino ex-60% fund to FDC is also gratefully acknowledged. AF was supported by a PhD fellowship from the Italian Ministry of University and Research (MIUR).

## Conflict of interest

The authors declare that the research was conducted in the absence of any commercial or financial relationships that could be construed as a potential conflict of interest.

## Publisher’s note

All claims expressed in this article are solely those of the authors and do not necessarily represent those of their affiliated organizations, or those of the publisher, the editors and the reviewers. Any product that may be evaluated in this article, or claim that may be made by its manufacturer, is not guaranteed or endorsed by the publisher.
